# Searching for lost nucleotides of the pre-RNA World with a self-refining model of early Earth

**DOI:** 10.1038/s41467-018-07389-2

**Published:** 2018-12-12

**Authors:** Nicholas V. Hud

**Affiliations:** NSF-NASA Center for Chemical Evolution, School of Chemistry and Biochemistry, Georgia Institute of Technology, Atlanta, GA 30332 USA

## Abstract

The nucleotides of RNA appear to be products of evolution. Experimental studies are showing that plausible proto-nucleotides can be formed in simulated early Earth environments. In turn, these results help to clarify the prebiotic processes that give rise to nucleotides.

The origin of the first nucleotides and the origin of RNA are related open questions, but they are not the same. That is, there may have been different nucleotides, and many more of them, on the prebiotic Earth than are found in extant RNA. The prebiotic synthesis of the RNA nucleotides is a tall order, and so is the prebiotic formation of RNA polymers (even with pre-formed nucleotides). Uncovering the origins of nucleotides and genetic polymers requires understanding the various chemical reactions that produce such molecules, whether or not (or how much) RNA nucleotides have evolved from earlier forms, and what prebiotic environments facilitate their formation.

## The possibility of RNA-first or RNA-later

In the search for the origins of nucleotides and RNA, there are two main camps, or schools of thought. In the RNA-first camp, RNA arose abiotically and is the first informational polymer of life, with its nucleotides and polymers produced entirely by geochemical reactions^1^. In the RNA-later camp, the extant RNA nucleotides are considered to be products of chemical and/or early biological evolution^2^. Both camps face substantial challenges in reconstructing the actual historical origin of nucleotides, but the challenges are quite different. The RNA-first proponents have an advantage in that they know exactly which molecules they seek to produce with model prebiotic reactions. However, they are limited by the chemical stability, reactivity, and functionality of the molecular subunits that comprise the extant nucleotides. Consequently, the RNA-first proponents must, understandably, sometimes rely on specific minerals (e.g., borate) or geological events (e.g., meteorite impacts) to control, guide, or initiate a particular reaction or reaction sequence^3,4^. The RNA-later proponents, on the other hand, can draw from a wider variety of molecular building blocks, including molecules with more favorable properties for spontaneous nucleotide formation. However, with this increased freedom comes the liability of becoming lost in the vast chemical space of possible prebiotic molecules without ever finding the nucleotides that initiated the evolutionary march toward RNA.

## Clues and challenges for prebiotic nucleotide synthesis

**Fig. 1:**
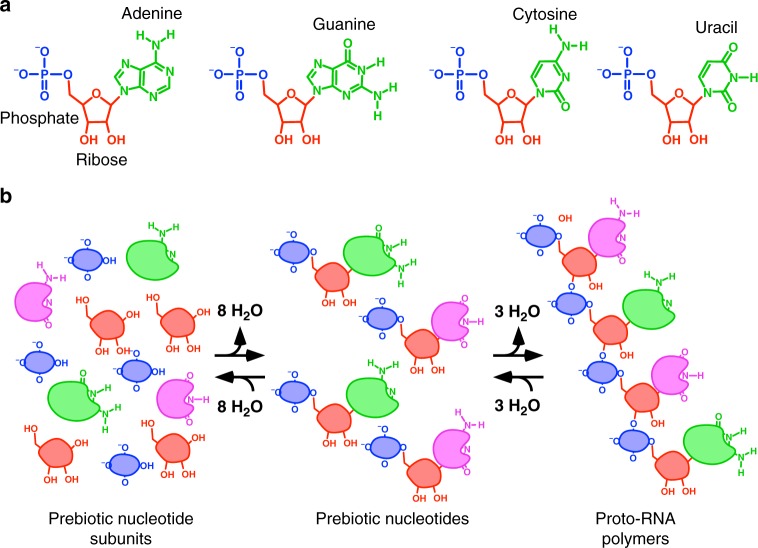
The RNA nucleotides and a hypothetical process for prebiotic nucleotide and proto-RNA formation. **a** The extant RNA nucleotides. The name of each nucleobase is given above its respective nucleotide. The phosphate and ribose subunits of the adenine nucleotide are labeled to emphasize the modular molecular structure of nucleotides. **b** Schematic representation of prebiotic nucleotide synthesis and polymerization. The subunits of the prebiotic nucleotides are shown as nondescript molecular entities because their identities are unknown. Some functional groups of the extant nucleotides are shown on the prebiotic nucleotide subunits to emphasize that some chemical and structural features of the earliest nucleotides may have been retained over the course of evolution due to the constraints imposed by a requirement for forward compatibility, such as the ability to form Watson-Crick, or similar, base pairs. The reversibility of nucleotide formation and polymerization, indicated by reverse arrows, emphasizes our hypothesis that early nucleotides and proto-RNA polymers would have had linkages that were more thermodynamically favored and less kinetically trapped compared to the extant nucleotides and nucleic acids^14^

The chemical structure of the extant RNA nucleotides is modular, with each nucleotide containing three molecular subunits: phosphate, ribose and a nucleobase (Fig. 1a). This general structure has long inspired chemists to hypothesize that the subunits were formed separately on the early Earth and subsequently joined together by dehydration condensation reactions^5^. That is, both the bond connecting phosphate to ribose and the bond between ribose and the nucleobase release a water molecule as they are formed (Fig. 1b). Similarly, the coupling of each nucleotide to a growing RNA polymer also generates a water molecule (Fig. 1b). Thus, based on Le Chatelier’s principle, nucleotide formation and polymerization are both more favored thermodynamically when subunit and nucleotide concentrations increase and the water concentration decreases (i.e., at low water activity).

Obviously, on the early Earth conditions of low water activity would have been prevalent on dry land. Additionally, evaporation of small bodies of water (e.g., rain puddles, ponds, tidal pools) would have concentrated dissolved nucleotide subunits during a dry season and perhaps even during the daylight hours of a diurnal cycle. Heating the dry surface would also promote nucleotide formation by providing the kinetic energy needed for condensation of co-deposited nucleotide subunits. This drying pond model for prebiotic nucleotide synthesis is simple, thermodynamically sound, and easy to imagine on the early Earth. Nevertheless, attempts to produce the extant nucleotides in laboratory models of such environments, by drying and heating mixtures of nucleotide subunits, have failed to produce nucleotides in meaningful amounts. In particular, drying and heating adenine, cytosine, guanine, and uracil with ribose does not result in bond formation between ribose and these nucleobases, or only in very low yields^5^.

## Wet–dry cycles and proto-nucleotide formation and evolution

My co-workers and I find the drying pond model to be the most plausible scenario for the prebiotic synthesis of nucleotides. We are also are proponents of the RNA-later scenario. We have hypothesized that ribose and the four nucleobases have evolved from related molecular subunits that constituted the earliest nucleotides (Fig. 1b)^2^. We hypothesize that the early nucleotide subunits reacted more easily with each other to form proto-nucleotides when dried and heated on the surface of the early Earth. Our experimental investigations of this hybrid drying-pond/RNA-later hypothesis have resulted in the identification of nitrogen-containing heterocycles (molecules similar in structure to the nucleobases of RNA) that spontaneously form nucleotide-like molecules in good yields when dried and heated with ribose or with other sugars^6,7^.

We also believe that the wet phases of wet–dry cycles played an important role in the synthesis and early evolution of nucleotides. It is unlikely that the subunits of the prebiotic nucleotides would have been formed and initially deposited in the same locations. In which case, hydration cycles would have been critical for mobilizing and bringing the subunits together. On a microscopic scale, model prebiotic nucleotide yields can increase when dried subunit mixtures are regularly rehydrated^8^, apparently because wet phases enable redistribution of unreacted molecules to more favorable locations/orientations for nucleotide formation during the next dry phase. Additionally, biological polymers function only in the hydrated state, so at some point water must be added to polymers of RNA (or proto-RNA) nucleotides. It is therefore likely that evolutionary pressures at the polymer level in the solution state, such as duplex stability and the ability to form catalytic structures^9^, set the prebiotic nucleotides on a path of incremental changes that eventually produced the extant nucleotides.

## Making laboratory experiments representative of early Earth

Results from our model laboratory experiments on prebiotic nucleotide synthesis are used to optimize future experiments and to provide a refined understanding of the environments on the early Earth that gave rise to nucleotides. For example, we first studied the reactions of ribose with alternative nucleobases at 100 °C because this high temperature was required to see any evidence of bond formation between ribose and adenine (an extant nucleobase^5^). However, we now are able to conduct most nucleotide synthesis experiments between 55° and 85 °C. Our recent discovery of more reactive model proto-nucleobases permits this less extreme temperature range. This reduction in temperature is important because the prebiotic building blocks (e.g., some sugars, amino acids, hydroxy acids) show rapid increases in degradation rates above 85 °C. Some model prebiotic reactions we study do not occur at temperatures below 55 °C. Significantly, temperatures between 55° and 85 °C were likely common on Earth’s surface at the time when life began^10^.

We are also examining the precursors of prebiotic nucleotide subunits for clues to the natural environment in which nucleotides first arose. In this regard, it seems indisputable that urea, the small molecule most often associated with urine, was a key component in the synthesis of prebiotic nucleotides. Urea is a precursor in the synthesis of numerous nitrogen-containing heterocycles, including the extant nucleobases and the heterocycles that we have found to readily form model prebiotic nucleotides. Specifically, these and other critically important prebiological compounds (e.g., amino acids) are formed when a concentrated solution of urea is placed in a sealed reaction vessel under a model prebiotic atmosphere^11^.

Although not obvious at first, we think that large regions of concentrated urea were common on the surface of the prebiotic Earth that were similar to today’s Bonneville Salt Flats of Utah. These urea flats are possible because urea is a major product of model prebiotic atmospheric reactions^12^, urea has a solubility of around 8 M in water (making it freely mobile in precipitation and runoff), and urea is a very stable molecule, which allows for its accumulation. Urea also forms water-free and low-water eutectic liquids with a number of organic compounds and inorganic salts. This property increases the availability of urea and other potentially important ingredients for nucleotide synthesis. For example, we have found that some urea-based liquids liberate phosphate from hydroxyapatite, a mineral that is essentially insoluble in water and whose lack of solubility had been viewed as an obstacle to phosphate incorporation into nucleotides of the early Earth^13^.

**Fig. 2:**
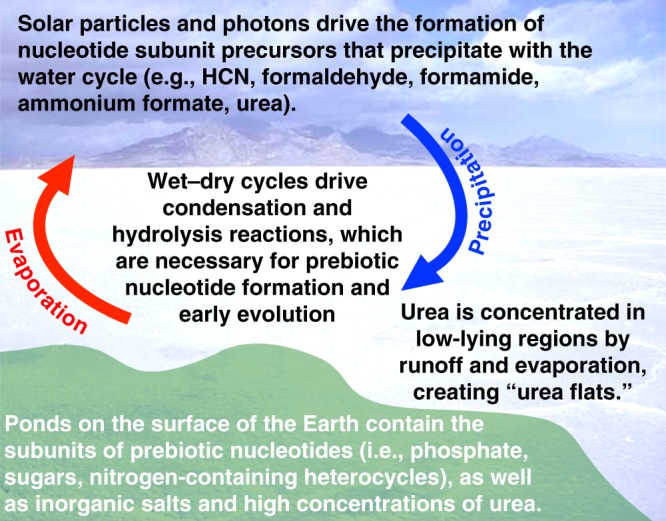
Illustration of an early Earth environment considered most favorable for nucleotide formation. From top to bottom: Precursors for the synthesis of the organic subunits of nucleotides are formed in the atmosphere of the early Earth with energy provided by photons and solar energetic particles (e.g., high energy protons from the young Sun^15^). These precursors include formaldehyde, HCN, the products of HCN reacting with water (i.e., formamide and ammonium formate), urea, as well as others not listed. Precipitation brings these molecules along with water to the surface of the Earth. Urea is concentrated in low-lying regions on the surface of the Earth due to its high production, chemical stability, solubility in water, and low volatility. Solutions containing high concentrations of urea, other dissolved organics, and phosphate provide the subunits of prebiotic nucleotides. These subunits form dehydration condensation bonds to create nucleotides during times of low water activity and elevated temperatures

Currently, based on the findings presented above, we conclude that the most favored environment for prebiotic nucleotide formation on the early Earth was a pond on dry land, with dissolved nucleotide subunits, and a substantial concentration of urea (Fig. 2). This pond would have been subjected to regular wet–dry cycles, and temperatures reaching as high as 85 °C. Nucleotide synthesis and polymerization occurred during hot-dry phases and early steps of chemical evolution occurred during cool-wet phases. The unmatched power of evolution eventually resulted in the emergence of extant RNA.

## On continuing the search

Our search for the origin of nucleotides has shown that the drying pond model naturally enables the prebiotic formation of nucleotides if we accept that the first nucleotides were different from extant nucleotides. Further experimental investigations will bring us continuously closer to identifying the earliest nucleotides that initiated the evolutionary march toward RNA. I am optimistic that such lost treasures of the pre-RNA World will be revealed in the future if we do not, as a research community, prematurely declare the origin of nucleotides and nucleic acids a solved problem.
